# Rare Presentation of a Low-Grade Spindle Cell Neoplasm in the Anterior Abdominal Wall

**DOI:** 10.7759/cureus.97977

**Published:** 2025-11-27

**Authors:** Rithik Naik Korra, Sonu Rahul Tej Gaddam, Nishal A Kumar, Asma Farheen Sheik, Samiya Saher, Jai Hind Reddy Nalla, Hema Manvi Koneru, Rashika R Ananthula, Ajay Goud Nattala, Nikhil Kumar Balagoni

**Affiliations:** 1 Internal Medicine, Osmania Medical College, Hyderabad, IND; 2 Radiology, Osmania Medical College, Hyderabad, IND; 3 General Internal Medicine, Andhra Medical College, Visakhapatnam, IND; 4 Internal Medicine, Rajiv Gandhi Institute of Medical Sciences, Adilabad, IND; 5 General Medicine, Bhaskar Medical College, Yenkepally, IND; 6 General Medicine, Osmania Medical College, Hyderabad, IND

**Keywords:** abdominal wall tumor, histopathology, soft tissue neoplasm, spindle cell neoplasm, surgical excision

## Abstract

Spindle cell neoplasms are morphologically distinct sets of mesenchymal tumors characterized by elongated, spindle-shaped cells arranged in fascicles. They may be benign or malignant and can arise in various anatomical locations. Their variable histological pattern and non-specific clinical features make the diagnosis difficult, particularly when they occur in rare sites such as the anterior abdominal wall.

We present a case of a 50-year-old male patient who presented with a progressively enlarging right lower abdominal mass over two years, recently becoming painful and ulcerated with purulent discharge. He had a history of a similar lesion over the right leg 20 years prior, which was excised and suspected to be an implantation dermoid. Examination revealed a 10 x 15 cm pedunculated, firm-to-hard, bosselated mass in the right lower anterior abdominal wall without regional lymphadenopathy. CT imaging demonstrated a multilobulated, heterogeneously enhancing soft tissue lesion in the subcutaneous plane (8.9 × 5.2 × 9.0 cm), without intra-abdominal extension and small umbilical hernia. Biopsy revealed a low-grade spindle cell neoplasm. Wide local excision with hernia repair was performed successfully. Histopathology confirmed the diagnosis, and the patient remains recurrence-free on follow-up.

Spindle cell neoplasms at uncommon sites, such as the anterior abdominal wall, may clinically mimic benign lesions, making diagnosis challenging. Imaging may be misleading, highlighting the importance of the tissue biopsy for definitive diagnosis. Early histopathological confirmation and complete surgical excision are critical for optimal outcomes in rare spindle cell neoplasms.

## Introduction

Spindle cell neoplasms are a heterogeneous group of tumors characterized histologically by elongated, spindle-shaped cells arranged in fascicles [1]. These lesions may arise from mesenchymal, epithelial, or neurogenic lineages and can exhibit benign, intermediate, or malignant behavior. The differential diagnosis is broad and includes fibromatoses, leiomyomas, dermatofibrosarcoma protuberans (DFSP), malignant peripheral nerve sheath tumors, and soft tissue sarcomas [2,3].

According to the 2020 World Health Organization (WHO) Classification of Soft Tissue and Bone Tumors, spindle cell lesions are categorized based on their differentiation into fibroblastic/myofibroblastic, smooth muscle, nerve sheath, vascular, or tumors of uncertain differentiation [4]. The term low-grade spindle cell neoplasm is not a distinct WHO-defined entity but rather a descriptive histopathologic diagnosis used when a tumor demonstrates spindle cell morphology without a clearly established lineage. In such cases, immunohistochemistry (IHC) is essential to identify the tumor’s origin. However, due to the unavailability of IHC testing in our case, the diagnosis was based on biopsy findings showing a low-grade spindle cell lesion, and management was guided by clinical and operative findings.

Low-grade spindle cell neoplasms of the anterior abdominal wall are exceedingly rare, likely due to the limited amount of mesenchymal tissue in this region. Their nonspecific clinical presentation and overlapping imaging characteristics often complicate diagnosis. On ultrasound, CT, or MRI, these lesions may appear as solid soft-tissue masses, resembling more common entities such as desmoid tumors, lipomas, sarcomas, or metastatic deposits. A study analyzing abdominal wall masses found that desmoid tumors accounted for 30%, followed by sarcomas (20%), metastases (18%), and lipomas (6%), underscoring the diagnostic diversity of lesions in this area [5]. Given this overlap, misdiagnosis is not uncommon; desmoid tumors are reportedly misidentified in up to 40% of cases, often mistaken for lipomas, and in rare instances, desmoid tumors of the chest wall have even been confused with breast cancer on clinical and imaging evaluation [6,7].

This case contributes to the limited literature on low-grade spindle cell neoplasms of the anterior abdominal wall and highlights several important points: the rarity of this presentation, the diagnostic uncertainty that arises in the absence of immunohistochemical confirmation, and the need to maintain a broad differential diagnosis when evaluating abdominal wall masses.

## Case presentation

A 50-year-old man presented to the outpatient department with a gradually progressive swelling over the right lower anterior abdominal wall for two years. The lesion was initially small and painless but showed more rapid growth during the second year. Two weeks before presentation, the swelling became painful and ulcerated, followed by purulent discharge. There were no associated constitutional or systemic symptoms. His past medical and family histories were unremarkable. The patient led a healthy lifestyle with no history of tobacco or alcohol use. However, the patient recalled a similar lesion on the right lower limb 20 years ago, which developed following trauma from a thorn and was surgically excised. It had been presumed to be an implantation dermoid.

**Figure 1:**
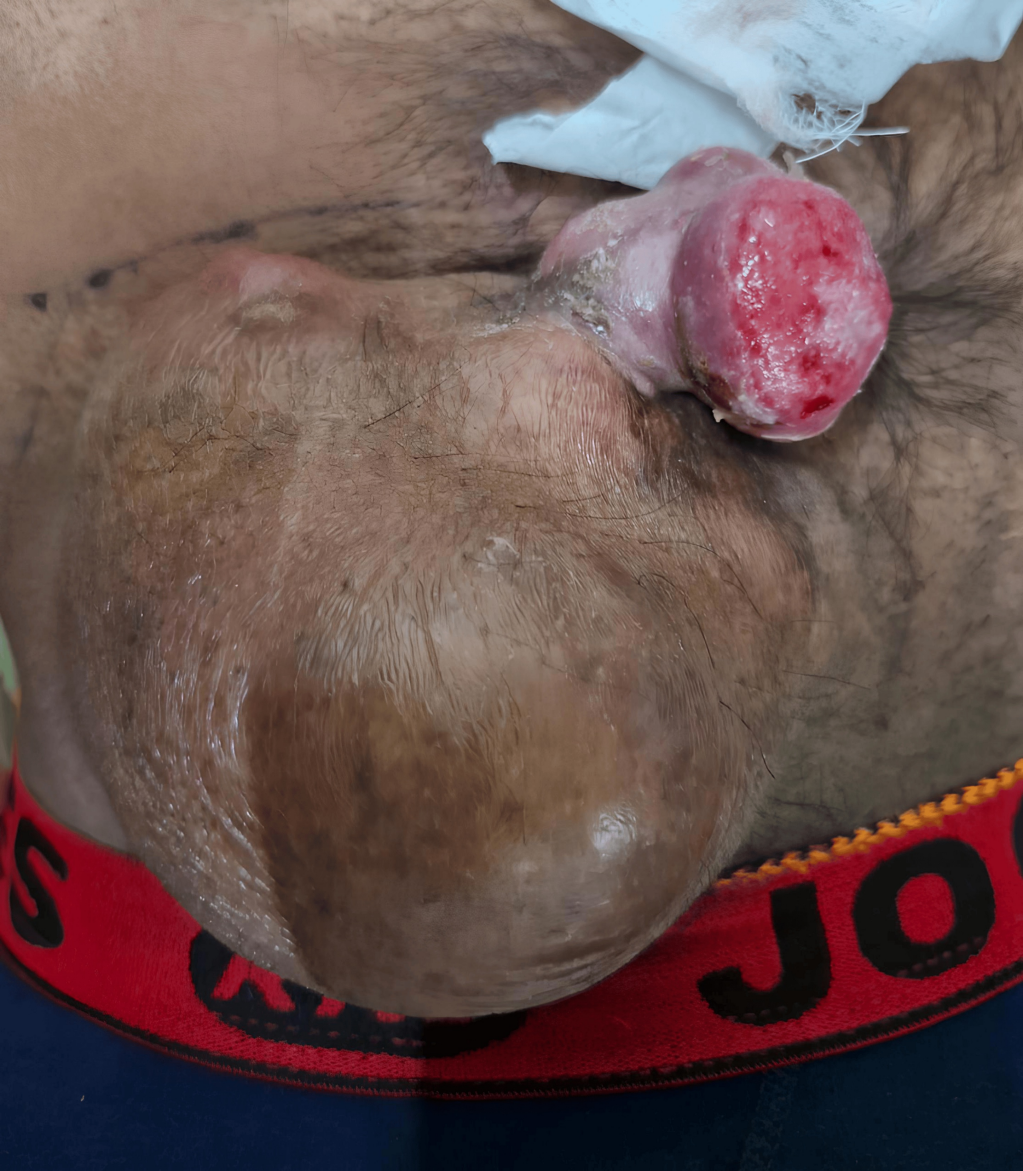
Gross image of pedunculated abdominal wall mass with ulceration and seropurulent discharge

On physical examination, the patient was afebrile and hemodynamically stable. Local examination revealed a pedunculated, nodular swelling measuring approximately 10 × 15 cm in the right lower anterior abdominal wall (Figure 1). The mass had a bosselated, irregular surface with ulceration and seropurulent discharge. It was firm to hard in consistency, non-tender, and mobile, and not adherent to the underlying muscle. The lesion had well-defined margins and became more prominent with leg raising. There was no cough impulse or regional lymphadenopathy.

**Figure 2:**
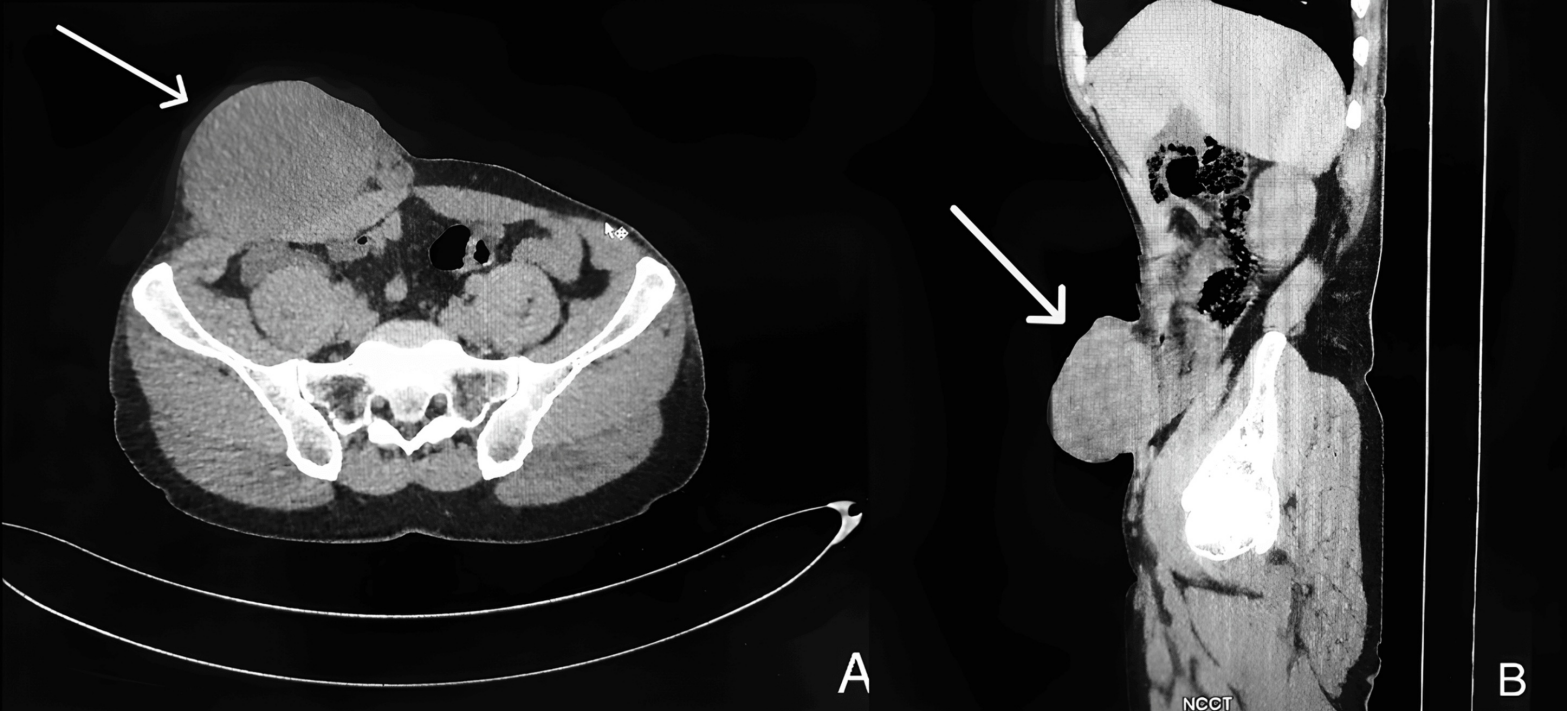
Axial and sagittal sections of plain CT showing a multilobulated, heterogeneously enhancing soft tissue density lesion, located in the subcutaneous planes of the right lower anterior abdominal wall, with no obvious intra-abdominal extension.

A contrast-enhanced computed tomography (CT) scan of the abdomen showed a multilobulated, heterogeneously enhancing soft tissue lesion measuring 8.9 × 5.2 × 9.0 cm located in the subcutaneous planes of the right lower anterior abdominal wall, causing indentation of the rectus abdominis muscle without intra-abdominal extension (Figure 2).

An incidental umbilical hernia was noted with a 1.82 cm defect containing herniating bowel loops. CT chest imaging and 2D echocardiography were unremarkable. Laboratory investigations, including complete blood count, renal and liver function tests, coagulation profile, and viral markers, were within normal limits (Table 1).

**Table 1:** Laboratory results during the course of hospitalization. Reference ranges for laboratory parameters were as follows: WBC (white blood cells): 4,000–11,000/cu.mm, Hb (hemoglobin): 13–17 g/dL (M), platelets: 150,000–450,000/cu.mm, urea: 15–40 mg/dL, creatinine: 0.6–1.2 mg/dL, total bilirubin: 0.2–1.2 mg/dL, direct bilirubin: 0.0–0.3 mg/dL, AST (aspartate aminotransferase): 10–40 U/L, ALT (alanine aminotransferase): 7–56 U/L, and ALP (alkaline phosphatase): 44–147 U/L.

Day	WBC (cu.mm)	Hb (g/dL)	Platelets (cu.mm)	Urea (mg/dL)	Creatinine (mg/dL)	Total Bilirubin (mg/dL)	Direct Bilirubin (mg/dL)	AST (U/L)	ALT (U/L)	ALP (U/L)
Day 1	7450	14.2	235,000	21	1.1	0.5	0.3	24	13	94
Day 2	9405	13.9	242,000	25	1.1	0.5	0.3	20	12	-
Day 3	7067	13.9	257,000	19	0.9	0.49	0.27	22	17	115
Day 5	4808	12.3	249,000	20	-	0.45	0.22	25	16	122
Day 7	5370	13.6	224,000	-	-	-	-	-	-	-
Day 8	-	-	-	-	0.92	0.46	0.2	30	17	103
Day 10	6723	14.1	233,000	22	-	-	-	-	-	-
Day 12	-	-	-	25	0.94	-	-	33	17	-
Day 13	6430	14.3	231,000	19	-	0.46	0.26	32	19	79
Day 15	5630	14.1	250,000	22	0.87	-	-	-	-	-

**Figure 3:**
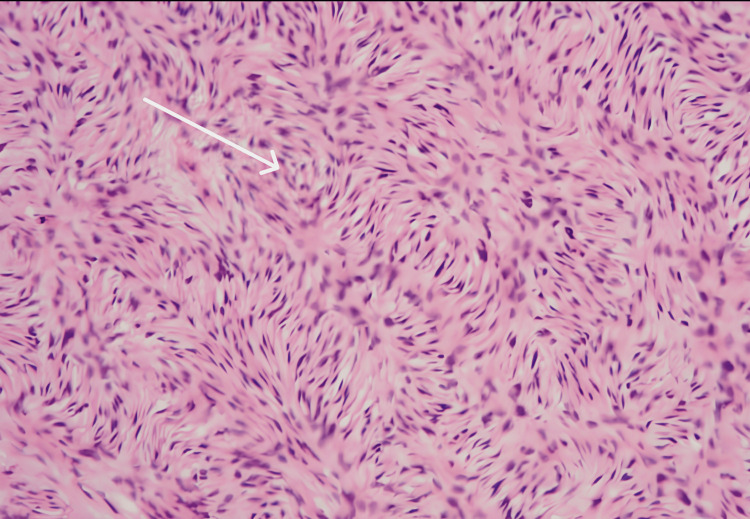
The hematoxylin and eosin (H and E) stained section showing sheets of spindle cells with mild to moderate anisonucleosis.

Fine-needle aspiration cytology (FNAC) of the lesion was suggestive of a mesenchymal neoplasm. A subsequent tru-cut biopsy revealed spindle-shaped cells with mild to moderate anisonucleosis, suggestive of a low-grade spindle cell neoplasm. The patient was referred to surgical oncology and underwent wide local excision of the abdominal wall mass along with repair of the umbilical hernia. The postoperative course was uneventful. Gross pathological examination showed linear tissue cores composed of spindle cells. Final histopathological analysis confirmed a low-grade spindle cell neoplasm (Figure 3). At the six-month follow-up, there was no evidence of recurrence, and the patient continues under routine oncological surveillance.

## Discussion

Spindle cell neoplasms originate from mesenchymal cells, which comprise the body’s connective tissue, including neural, fibroblastic, myofibroblastic, epithelial, and vascular components. Based on their biological behavior, they are categorized as benign, intermediate (locally aggressive or rarely metastasizing), and malignant types [8]. According to the 2020 WHO Classification of Soft Tissue and Bone Tumors, a low-grade spindle cell neoplasm is not recognized as a distinct entity but rather used as a descriptive histopathologic diagnosis when the tumor demonstrates spindle cell morphology without a clearly established lineage [9]. Spindle cell tumors of the abdominal wall are extremely rare, as most soft tissue neoplasms occur in the extremities. When they arise in unusual locations, particularly in patients without traditional risk factors, diagnosis can be delayed or misclassified. The etiology of soft tissue tumors is multifactorial, involving an interplay of prior trauma or surgery, genetic factors, and environmental causes such as viral infections, irradiation, and immunodeficiency disorders [10]. Certain familial syndromes like Gardner’s syndrome (desmoid tumors), neurofibromatosis (peripheral nerve sheath tumors), and Li-Fraumeni syndrome (sarcomas) are also associated with an increased risk [11].

Soft tissue tumors usually appear in the extremities, although they are very uncommon in the abdomen [12]. In our case, the presentation of a spindle cell neoplasm on the anterior abdominal wall in a 50-year-old male patient was atypical. The clinical course was slow and insidious, with ulceration and discharge developing later, prompting further evaluation. Based on clinical examination alone, differential diagnoses included implantation dermoid, epidermal inclusion cyst, DFSP, desmoid-type fibromatosis, and low-grade soft tissue sarcoma [2,12]. Although the patient’s previous history of a similar lesion suggested a benign etiology, the evolution of symptoms raised concern for malignancy.

Cross-sectional imaging, specifically contrast-enhanced CT, played an important role in delineating the lesion’s subcutaneous location, size, and relationship to adjacent structures. It also ruled out intra-abdominal or retroperitoneal extension and incidentally revealed a small umbilical hernia, which was repaired during surgery. Imaging revealed heterogeneous enhancement and multilobulated contours suggestive of a neoplastic process but remained non-specific. In such cases, radiological findings should always be interpreted alongside histopathology to avoid diagnostic pitfalls [10]. Both CT and MRI are valuable in defining tumor size, margins, and staging of soft tissue tumors but cannot reliably distinguish them from mimics like desmoid fibromatosis, lipomas, or metastases.

Histopathology remains the gold standard for the diagnosis and grading of soft tissue tumors [10]. In atypical presentations, the integration of imaging, biopsy, and histology provides critical guidance for surgical planning and oncological decision-making. FNAC, a minimally invasive procedure, shows good concordance with histology in most cases. However, due to overlapping cytologic features among different spindle cell tumors, core needle biopsy provides better architectural details and cellularity for grading and management planning. This distinction is particularly relevant in differentiating low-grade neoplasms from more aggressive sarcomas [2-4]. Immunohistochemical markers play an important role in differentiating and grading tumors. Unfortunately, it could not be performed in our case due to unavailability of testing. As a result, the diagnosis was based solely on morphology, and the tumor was classified as a low-grade spindle cell neoplasm. This reflects a real-world diagnostic challenge often encountered in resource-limited settings.

A similar case was reported by Kim et al., in which a retroperitoneal spindle cell tumor presented with nonspecific abdominal pain and overlapping radiologic features, requiring histopathologic and immunohistochemical confirmation for accurate diagnosis [13]. As with our case, definitive diagnosis required a multidisciplinary approach, and complete surgical excision led to an excellent outcome. A review of the literature shows that early recognition, complete surgical excision with negative margins, and regular follow-up are key to optimal outcomes, especially for low-grade lesions. In contrast, high-grade tumors or those with recurrence, particularly those harboring newly described molecular alterations such as EWSR1-NACC1 or FUS-NACC1 fusions, may warrant molecular profiling, IHC, and adjuvant therapy [2,14,15].

Adjuvant treatment choices depend largely on the tumor’s grade, size, surgical margins, and recurrence risk. Both the NCCN (2024) and ESMO guidelines emphasize that the cornerstone of management is complete surgical removal with clear margins. Radiotherapy is often recommended for high-grade sarcomas to reduce local recurrence, while chemotherapy with agents such as doxorubicin or ifosfamide may be considered for larger, recurrent, or metastatic tumors, though its role in low-grade lesions remains limited. Recent advances, including targeted therapies like pazopanib or trabectedin, immunotherapy, and newer modalities such as high-intensity focused ultrasound, show promise in certain sarcoma subtypes, though their role in spindle cell tumors is still being explored. Importantly, imaging features such as heterogeneity or multilobulation often correlate with histopathological indicators of aggressiveness, including mitotic activity and nuclear atypia. This radiologic-pathologic correlation is crucial in guiding prognosis and tailoring adjuvant modalities. Both NCCN and ESMO also highlight the importance of a multidisciplinary tumor board discussion, where surgeons, oncologists, radiologists, and pathologists collaborate to design the most appropriate treatment plan for each patient [16-18].

Our case aligns with these conclusions, underscoring the importance of maintaining a broad differential diagnosis, judicious use of imaging and biopsy techniques (including FNAC and core biopsy), complete surgical removal with clear margins, and vigilant postoperative surveillance, with no current indication for adjuvant therapy.

## Conclusions

This case underscores the importance of maintaining a broad differential diagnosis and emphasizing early histopathological evaluation when assessing anterior abdominal wall masses. Clinically indolent or benign-appearing lesions may conceal rare entities such as low-grade spindle cell neoplasms, leading to potential delays in diagnosis and management. In our case, the radiologic findings were inconclusive, highlighting the value of early tissue sampling in achieving diagnostic clarity. Despite the absence of immunohistochemical testing, a core needle biopsy provided sufficient information for appropriate surgical planning. Complete excision with negative margins resulted in an excellent outcome, reinforcing surgery as the cornerstone of management for localized, low-grade lesions.

This case further reinforces the need for heightened clinical vigilance and a multidisciplinary approach when evaluating soft tissue masses in atypical locations. Integrating clinical, imaging, and histopathological findings remains essential to ensure timely diagnosis, effective treatment, and improved patient outcomes.
